# Case Report: Remarkable and sustained remission of an anaplastic thyroid carcinoma patient to the combined treatment of multimodal radiotherapy, anlotinib and toripalimab

**DOI:** 10.3389/fonc.2025.1491918

**Published:** 2025-05-21

**Authors:** Yurou Xing, Xuehu Wu, Zhihui Li, Xin Wu

**Affiliations:** ^1^ Thoracic Oncology Ward, Cancer Center, West China Hospital, Sichuan University, Chengdu, Sichuan, China; ^2^ Department of Oncology, Minda Hospital of Hubei Minzu University, Enshi, China; ^3^ Division of Thyroid Surgery, Department of General Surgery, West China Hospital, Sichuan University, Chengdu, China; ^4^ Head and Neck Oncology Ward, Cancer Center, West China Hospital, Sichuan University, Chengdu, China

**Keywords:** anaplastic thyroid carcinoma, antiangiogenic drug, multimodal radiotherapy, immunotherapy, sustained remission anaplastic thyroid carcinoma

## Abstract

Anaplastic thyroid carcinoma (ATC) is an infrequent malignant tumor that has a high death rate and a poor prognosis. The therapeutic methods for ATC include traditional surgery, chemoradiotherapy and targeted therapy. However, the effect of ATC treatment is not ideal. We report a patient with ATC who developed rapid local recurrence and multiple systemic metastases after radical thyroidectomy. After multimodal radiotherapy, antiangiogenic therapy and immunotherapy, the patient’s tumors shrunk significantly and achieved long-term control. The therapeutic effect was remarkable. These findings provide a viable new treatment choice for ATC patients in the future.

## Introduction

Anaplastic thyroid carcinoma (ATC) is an extremely aggressive thyroid tumor that accounts for approximately 1-2% of all thyroid cancers (1). ATC is most common in older adults and women (2, 3). ATC often manifests as a mass in the neck, and symptoms, such as hoarseness, difficulty swallowing, difficulty breathing, etc (4). Imaging examinations of ATCs include cervical ultrasound, CT and positron emission tomography (PET)-CT. The methods used to obtain pathological tissue include fine needle puncture, surgical biopsy, etc. ATC has a high probability of adjacent tissue invasion and metastasis, with the main type of metastasis usually occurring in the lungs, followed by bone (1). ATC has a high fatality rate, the median survival time is only 3–6 months, and the one-year overall survival (OS) rate is approximately 20% (5). However, there is no optimal treatment standard for ATC patients. Traditional treatment modalities include surgery, radiotherapy, and chemotherapy (6), but the therapeutic effect is limited. Targeted therapy for driver gene mutation and immunotherapy are new therapeutic options (7, 8). We report that in an advanced ATC patient who received multimodal radiotherapy, anlotinib (an antiangiogenic drug) and toripalimab (a PD-1 antibody), the tumors were effectively controlled for more than two years.

## Case presentation

**Figure 1:**
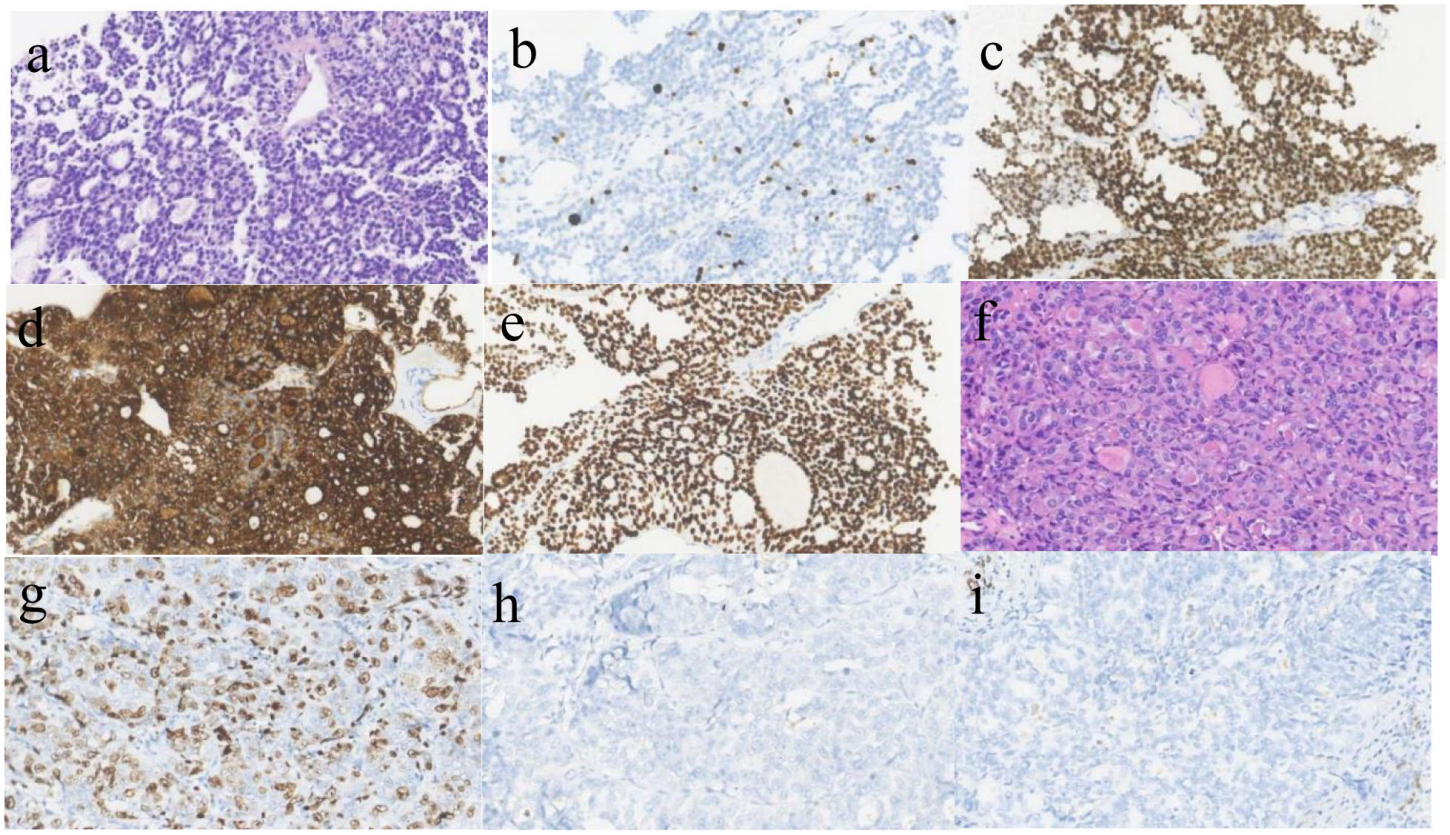
Pathological image **(a)** follicular carcinoma of thyroid.(HE*400)The follicular structure of the thyroid gland was observed, some follicles contain gum. **(b)** Ki-67 is expressed in FTC, about 10%(x400); **(c)**. PAX-8 is positive in FTC(x400); **(d)**. TG is positive in FTC(x400); **(e)** TTF-1 is positive in FTC(x400); **(f)**. Anaplastic thyroid carcinoma(HE*400),The tumor cells were large, the nuclei were moderately and severely deformed, and the mitotic images were common; **(g)**. Ki-67 is expressed in ATC, about 70%(x400); **(h)**. TG is negative in ATC(x400); **(i)** TTF-1 is negative in ATC(x400).

**Figure 2:**
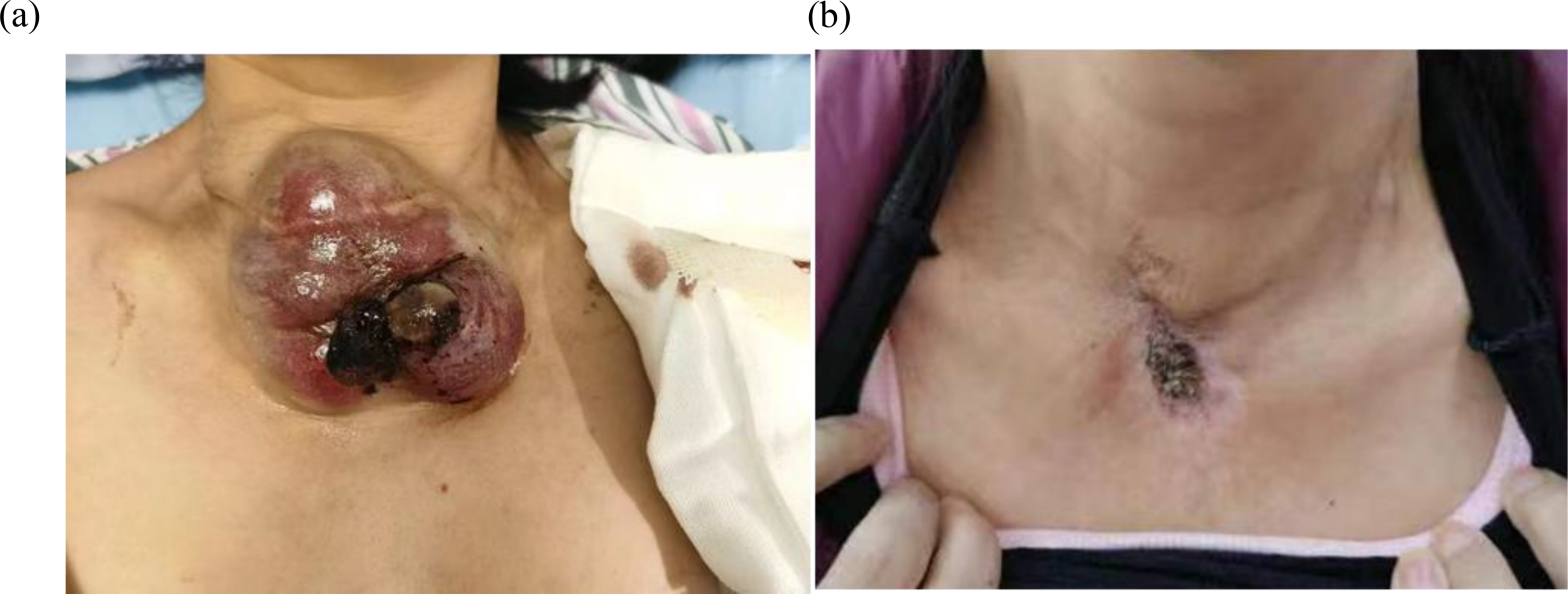
**(a)** The neck mass, about 10cm*8cm*6cm in size, locally ruptured and bled in July 2021. **(b)** After accepting radiotherapy, anlotinib and immunotherapy, the patient’s neck mass shrank, as shown above in July 2023.

A 43-year-old woman with recurrent thyroid cancer after surgery was admitted to our hospital. She had no other special medical history and no family history of tumors. In 2013, the patient found a neck mass, and an ultrasound examination at a local hospital indicated a thyroid tumor. Total thyroidectomy plus cervical lymph node dissection was subsequently performed. Postoperative pathology revealed follicular thyroid carcinoma, and the tumor infiltrated the soft tissue surrounding the thyroid gland and was TG (+), TTF-1(+), PAX8(+), Calcitonin (-), CgA (-), Syn (-), PTH (-), and Ki- 67(+,10%) (**Figures 1a-e**). She received two rounds of iodine-131 therapy after surgery. She did not have regular review and follow-up after the operation. The patient underwent CT examination in June 2021 because of a growing mass on the neck, which revealed tumor recurrence. The patient came to our hospital for further treatment. A physical examination of the patient’s neck revealed a mass of approximately 10 cm*8 cm in size, hard quality, and poor mobility (**Figure 2a**). The surface of the mass was partially broken, resulting in bleeding. A CT scan at our hospital (2021-6-24) revealed that the cervical and mediastinal lymph nodes were enlarged and the larger one measured about 8.3cm x 4.3cm. A bone scan revealed multiple bone metastases on the right side of the pelvis. The pathological findings of the cervical mass puncture included TTF-1(-), PAX8(-), calcitonin (-), TG (-), PTH (-), CgA (-), Syn (-), CK (+), P53(+), CD117(-), CD5(-), P63(focal +), Ki-67(+, 70%), PDL1(+; TPS:15%, CPS:20), supporting for thyroid cancer (**Figures 1f-i**). Genetic tests detected a NRAS (p.Q61R) mutation.

**Figure 3:**
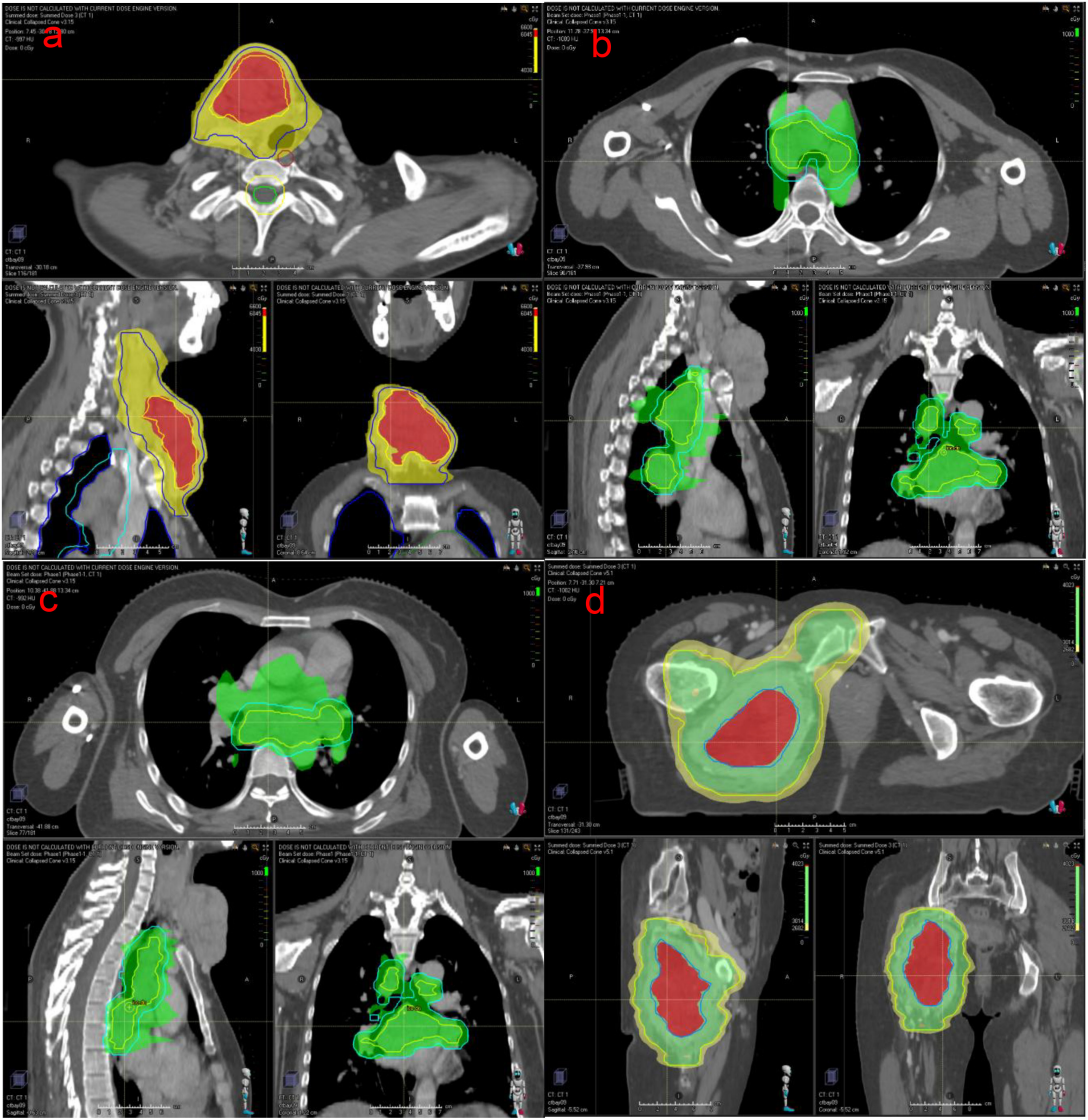
The map of the patient’s radiotherapy target volume. **(a)** neck; **(b, c)** chest; **(d)** pelvis.

**Figure 4:**
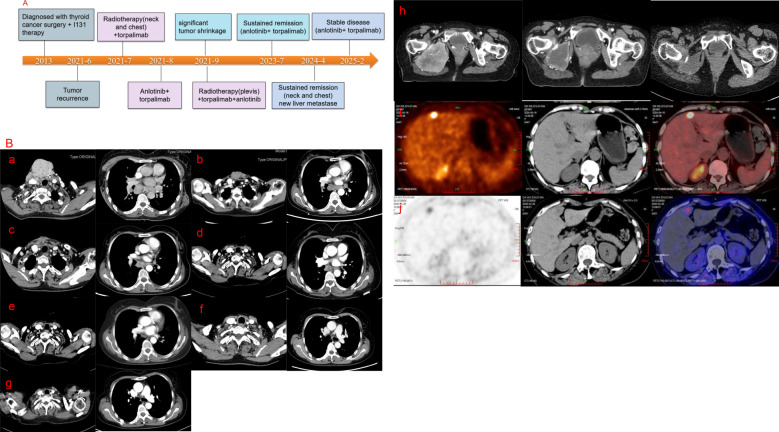
**(A)** Timeline of treatment. **(B)** A series of CT images of the patient. a-g, CT images of the patient’s neck and chest. (a) June 2021 (b) September 2021 (c) November 2021 (d) April 2022 (e) July 2023 (f) January 2024 (g) February 2025. (h) CT images of the bone metastasis, and the examination time from left to right is September 2021, November 2021, and April 2022, respectively. Figure (i) shows the PET-CT image of the patient’s liver metastase in April 2024.Figure (j) shows the PET-CT image of the patient’s liver metastase in February 2025.

After multidisciplinary discussion in the departments of thyroid surgery, oncology and radiotherapy, and due to the large size of the patient’s tumor, multiple metastases and poor prognosis, surgical treatment was not possible, and combined immunotherapy with multimodal radiotherapy was recommended. The patient received multimodal radiotherapy in our hospital beginning July 6, 2021. The specific radiation prescription is 4000 cGy (tumor boost, the single dose was 400cGy) and 3000 cGy (the neck mass, the single dose was 300cGy) in 10 fractions and 1000cGy in 5 fractions to metastatic lymph nodes in the thoracic mediastinum (the single dose was 200cGy) using an Image Guide Radiotherapy (IGRT) (**Figures 3a-c**). The patient began to use toripalimab at a dose of 240 mg every three weeks beginning in July 2021. In August 2021, the neck mass decreased, bleeding stopped, and the patient began taking oral anlotinib (12 mg, once a day). The patient had a mild increase in blood pressure after receiving anlotinib, with a systolic blood pressure of about 150mmHg. Her blood pressure returned to normal after treatment with antihypertensive drugs. Side effects such as bleeding, nausea, and leukopenia were not observed, and liver and kidney function were normal in the patients receiving this combination therapy. At the same time, the patient had been using bisphosphonates to prevent adverse events such as fractures that could result from tumor bone metastasis. In September 2021, the patient underwent a CT examination: the neck and mediastinal lymph nodes were enlarged and significantly smaller than before, the largest of which was about 5.3cm x 2.6cm. The efficacy evaluation revealed a partial response. Pelvic CT of the patient revealed multiple bone destructions of the right femur and pelvis, accompanied by the formation of a soft tissue mass about 8.5cm*6cm in size. The patient was treated with radiation therapy due to hip pain and difficulty walking, and the applied dose was 6000 cGy (tumor boost area, the single dose was 400cGy) and 4500 cGy (pelvic mass, the single dose was 300cGy) in 15 fractions via IGRT in september 2021(**Figure 3d**). Her tumors gradually decreased in size with subsequent immunotherapy and antiangiogenic therapy (the cervical mass is shown in **Figure 2b**). Multiple systemic metastases have been well controlled. In April 2024, the patient underwent PET-CT, which revealed a new metastatic tumor in the liver measuring 1.5cm by 1.2cm. Because of the high risk of puncture, it is recommended that the patient be followed up dynamically and continue with the original regimen. Reexamination in 2025 showed no significant change in the liver lesion, and the lesions in the chest and neck had been well controlled. The patient is still receiving treatment with anlotinib and toripalimab. The CT results of the patients’ repeated visits are shown in the figure below. (**Figure 4**).

## Discussion

Although the incidence of ATC is not high, it accounts for the majority of thyroid cancer deaths (9, 10). In terms of staging, all ATC cases are stage IV, and half of ATC patients present with disease with extensive systemic metastasis (11, 12). The treatment of advanced ATC should be discussed in a multidisciplinary manner to develop a comprehensive treatment strategy. For resectable ATC, the preferred treatment modality is surgery (13). Radiotherapy plus chemotherapy with taxanes, anthracyclines, and platinum has been shown to prolong the survival of ATC patients (6, 14). Our patient did not receive chemotherapy because of her poor general condition. Dabrafenib and trametinib (BRAF/MEK inhibitors) should be recommended for BRAF V600E-positive ATCs (15). For advanced ATC patients with NTRK or RET mutations, TRK inhibitors (enrectinib, larorectinib) and RET inhibitors(selpercatinib) may be considered (16). This patient is not a candidate for targeted therapy because there are currently no drugs that target the RAS mutation.

As one of the local treatment methods, radiation therapy can reduce the tumor volume and prolong the survival of patients. In recent years, radiation therapy has become more and more accurate, which greatly protects normal tissues. For huge masses in the neck and pelvis, we applied high-dose field-in-field radiotherapy. High doses of radiotherapy can effectively kill tumor cells and is related to prolonged survival. In a study of 104 ATC patients, the median OS for patients receiving ≥60 Gy radiotherapy reached 10.6 months (17). Pezzi et al. analyzed 1288 patients with ATC using data from the National Cancer Center database and confirmed that patients receiving a higher (60–75 Gy) treatment dose had improved survival compared to those receiving a lower (45-59.9 Gy) treatment dose (18). Our patients received a multimodal radiotherapy. As the tumor increases in size, its internal blood circulation deteriorates, and even the central part may become necrotic, resulting in an increase in oxygen- depleted cells that are less sensitive to radiation (19). The method of large segmentation radiotherapy has a significant effect on the treatment of large masses with reduced central sensitivity. In the design of neck and pelvic cavity radiotherapy plans for this patient, we increased the dose in the hypoxic area of the tumor center, which effectively improved the local control rate. In the neck, there are important tissues such as large blood vessels, esophagus and airway, and in the pelvic cavity, there are femoral head, hip joint and intestine, etc. Our case shows that the field-in-field treatment (4500cGy/300cGy/15F in the peripheral area and 6000cGy/400cGy/15F in the central area of the tumor) was applied safely in our case and should be considered to be tested in a bigger patient cohort. We used low dose radiotherapy for mediastinal lymph nodes and the patients also received immunotherapy. ATC has more infiltrating lymphocytes and a higher positive expression rate of PD-L1. Immunotherapy has brought hope to these patients (20). In one study, patients with locally advanced or metastatic unresectable ATC who were treated with an immune checkpoint inhibitor (pembrolizumab or nivolumab) had a robust sustained response (21). In another study about spartalizumab, the 1-year survival rate of PDL-1-positive patients is as high as 52.1% in ATC patients (22). Low dose radiotherapy combined with immunotherapy play a remarkable role in increasing the sensitivity to radiotherapy and the effect of systemic therapy. Preclinical studies have confirmed that low-dose radiotherapy combined with immunotherapy can improve tumor immune microenvironment, increase T cell infiltration in tumor area and play a powerful anti-tumor killing effect (23). The combination of low-dose radiotherapy and immunotherapy can activate a variety of innate and adaptive immune pathways that play an antitumor role in tumors with low infiltration of immune cells (24). Combined with immunotherapy and anti-angiogenic therapy, our patient received low dose radiotherapy (1000cGy/200cGy/5F) to the metastatic mediastinal lymph nodes and achieved good results.

Targeted therapy can increase the efficacy of immunotherapy and anti-tumor effects. Anlotinib (a multi-target inhibitor that inhibits VEGFR, FGFR, PDGFR, and c- Kit) can significantly inhibit the growth and metastasis of ATC cells and induce the apoptosis of thyroid cancer cells (25). In addition, anlotinib can reduce the permeability of new blood vessels and induce the normalization of tumor blood vessels (26, 27). A study of 25 patients with advanced ATC confirmed an optimal ORR of 60% and a median PFS of 5.7 months for anlotinib combined with chemotherapy (28). Moreover, anti-angiogenic therapy combined with immunotherapy has been shown to have a synergistic anti-tumor effect. The normalization of tumor blood vessels can promote the infiltration of immune cells, transform an immunosuppressive microenvironment, and improve the effect of immunotherapy (29). A prospective phase 2 study (ATLEP trail) explored the efficacy of combination therapy with lenvatinib and pembrolizumab in 27 patients with metastatic ATC. The study confirmed that the best overall response rate of this combination therapy in the treatment of ATC within 2 years was 51.9% PR (14/27) and 44.4% SD (12/27). The median PFS of ATC patients was 9.5 months, and the median OS was 10.25 months (30). In our case, when the patient’s neck mass had shrunk significantly and the bleeding had stopped, we added anlotinib. The patient achieved long-term remission with the combination of anlotinib and toripalimab. Our case shows that the combination of anlontinib and toripalimab is highly effective, which further supports the rationale for combining immunecheckpoint inhibitors with VEGFR inhibitors.

In brief, this is the first time that new therapeutic strategies such as multimodal radiotherapy (including different dose segmentation of the local mass, also known as field-in-filed radiotherapy, and low dose radiotherapy of the chest metastatic tumors) combined with immunotherapy and targeted therapy have been applied to the treatment of ATC. This provides feasible new therapeutic modalities for the treatment of ATC.

## Conclusion

ATC is one of the most aggressive and lethal types of thyroid malignancies. We report a case of ATC treated with multimodal radiotherapy, anlotinib and toripalimab immunotherapy. The patient’s tumors were controlled for more than 3 years. Such good curative effects are quite rare in clinical practice. The results of our case using multimodal radiotherapy to treat ATC proved to be highly efficient and safe, which still needs to be actively explored. Multimodal radiotherapy, immunotherapy and anti- angiogenesis therapy have synergistic value. Our results provide clinicians with additional references for the treatment of ATC.

## Data Availability

The raw data supporting the conclusions of this article will be made available by the authors, without undue reservation.
