# Correction: Case Report: Remarkable and sustained remission of an anaplastic thyroid carcinoma patient to the combined treatment of multimodal radiotherapy, anlotinib and toripalimab

**DOI:** 10.3389/fonc.2025.1649567

**Published:** 2025-07-03

**Authors:** Yurou Xing, Xuehu Wu, Zhihui Li, Xin Wu

**Affiliations:** ^1^ Thoracic Oncology Ward, Cancer Center, West China Hospital, Sichuan University, Chengdu, Sichuan, China; ^2^ Department of Oncology, Minda Hospital of Hubei Minzu University, Enshi, China; ^3^ Division of Thyroid Surgery, Department of General Surgery, West China Hospital, Sichuan University, Chengdu, China; ^4^ Head and Neck Oncology Ward, Cancer Center, West China Hospital, Sichuan University, Chengdu, China

**Keywords:** anaplastic thyroid carcinoma, antiangiogenic drug, multimodal radiotherapy, immunotherapy, sustained remission anaplastic thyroid carcinoma

In the published article, there was a mistake in the author affiliation.

Author Xuehu Wu was erroneously assigned to affiliation “Division of Thyroid Surgery, Department of General Surgery, West China Hospital, Sichuan University, Chengdu, China”. This affiliation has now been removed for author Xuehu Wu.

Affiliation “Department of Oncology, Minda Hospital of Hubei Minzu University, Enshi, China” was omitted for author Xuehu Wu. This affiliation has now been added for author Xuehu Wu.

Author Xin Wu was erroneously assigned to affiliation “Department of Oncology, Minda Hospital of Hubei Minzu University”. This affiliation has now been removed for author Xin Wu.

The authors apologize for these errors and state that this does not change the scientific conclusions of the article in any way. The original article has been updated.

